# Assembly information services in the European Nucleotide Archive

**DOI:** 10.1093/nar/gkt1082

**Published:** 2013-11-08

**Authors:** Nima Pakseresht, Blaise Alako, Clara Amid, Ana Cerdeño-Tárraga, Iain Cleland, Richard Gibson, Neil Goodgame, Tamer Gur, Mikyung Jang, Simon Kay, Rasko Leinonen, Weizhong Li, Xin Liu, Rodrigo Lopez, Hamish McWilliam, Arnaud Oisel, Swapna Pallreddy, Sheila Plaister, Rajesh Radhakrishnan, Stephane Rivière, Marc Rossello, Alexander Senf, Nicole Silvester, Dmitriy Smirnov, Silvano Squizzato, Petra ten Hoopen, Ana Luisa Toribio, Daniel Vaughan, Vadim Zalunin, Guy Cochrane

**Affiliations:** European Molecular Biology Laboratory, European Bioinformatics Institute, Wellcome Trust Genome Campus, Hinxton, Cambridge CB10 1SD, UK

## Abstract

The European Nucleotide Archive (ENA; http://www.ebi.ac.uk/ena) is a repository for the world public domain nucleotide sequence data output. ENA content covers a spectrum of data types including raw reads, assembly data and functional annotation. ENA has faced a dramatic growth in genome assembly submission rates, data volumes and complexity of datasets. This has prompted a broad reworking of assembly submission services, for which we now reach the end of a major programme of work and many enhancements have already been made available over the year to components of the submission service. In this article, we briefly review ENA content and growth over 2013, describe our rapidly developing services for genome assembly information and outline further major developments over the last year.

## INTRODUCTION

The European Nucleotide Archive (ENA) is the European resource in which the world’s public domain sequencing data are maintained. ENA was established in the early 1980s to serve the scientific community and continues to provide a critical foundation for the global bioinformatics data infrastructure. ENA content covers a spectrum of data classes, from raw sequence reads, through read alignments and assembled sequences to functional annotation, along with various levels of descriptive information relating to these data. As a member of the International Nucleotide Sequence Database Collaboration (INSDC) (1), ENA operates routine data exchange with its partner resources in the USA (the National Center for Biotechnology Information) (2) and Japan (the DNA Databank of Japan) (3) and actively promotes free and unencumbered access to sequence data.

Three broad classes of activity define the work of the ENA team. These are the capture of sequence data from the scientific community, including the provision of submission tools and services, the efficient management and safe storage of the data and the presentation of the data to the research community, through search, retrieval and analysis services.

In this article, we review briefly ENA content and growth over 2013. We then describe our rapidly developing services in the area of genome assembly information. Finally, we outline further major developments over the year.

## CONTENT AND GROWTH

ENA content has continued to grow in volume and diversity through 2013 across the spectrum of its data classes. At the time of writing, ENA contains over 20 000 sequencing studies, over 570 trillion base pairs and over 18 000 assembled genomes. The majority of these genome assemblies have been submitted in 2013, indicating a broad use of new sequencing and assembly methods. Notable new datasets include data from the *Oryza sativa* Japonica Group (HAAQ01000001–HAAQ01000460) (4), a dataset from *Leishmania braziliensis* (HG323603) (5), important comprehensive third party annotations from the MiRBase (4) and SRPDB (6) projects, sequence datasets from metagenomics studies such as a study of the relationship between phytoplankton and bacterioplankton in the North Sea (ERP001227) (7) and a study on the human gut metagenome of 3-month-old infants (ERP001038) and a large number of important new genome assembly datasets, including reads, contigs and scaffolds from wheat (PRJEB3955), contigs and scaffolds from *Picea abies* (Norway spruce) (PRJEB1822) (8), contigs from camel (PRJEB407) (9) and 378 novel clinical isolate assemblies from the human distal gut (PRJEB674–PRJEB1046).

## ASSEMBLY INFORMATION SERVICES

### Changing landscape

**Figure 1.**
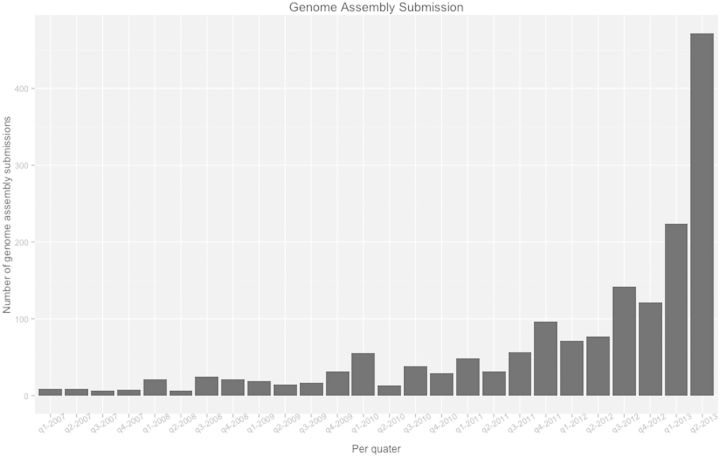
The figure presents the number of genome assembly submissions over time.

The archiving of genome assembly information has been a major function of ENA for many years. Early in the resource’s history, it was possible for all genome assembly data to undergo manual scrutiny under the eyes of scientific curation staff with each assembly being loaded manually into production databases. At the same time, the data submitter, typically an expert in assembly methods, and the curator were able to maintain a conversation around the details of the assembly in question, such that information was available to inform the way in which the assembly was structured within ENA. In the last 2 years, however, assembly submission has undergone dramatic growth. This growth represents both data volume increases (greater volume of sequence data per submission due to higher coverage sequencing and larger and more complex target genomes) and an increase in the number of assemblies being submitted (see Figure 1), particularly in relation to prokaryote data where we have witnessed a rise from around 30–40 submissions per quarter to over 450 in one recent quarter. Behind this growth is a dramatic shift in the availability and distribution of sequencing capacity from a comparatively small number of expert groups, to diverse facilities often with limited informatics capacity. These latter groups, which are typically in the clinical isolate sequencing and public health monitoring domains, provide data central to the completeness and usability of ENA, but require very different submission services and interfaces to those of our traditional research community.

We face three challenges in the area of assembly data: first, the complexity and diversity of assembly datasets challenge the capabilities of our submission, discovery and retrieval services for these data. Second, the number of submissions requires us to support workflows with no manual intervention on the part of ENA curation staff. Third, as a result of the size and complexity of genomes now being tackled (*Hordeum vulgare subsp. vulgare*, e.g. comprises over 2 million contigs, *Camelus bactrianus* 0.8 million, *P. abies* 11.3 million and wheat 11 million), a revision of the various data structures that are used to manage assembly data to support this scaling is required.

### New assembly data types

As part of our redevelopment, we have introduced a number of new data structures and have extended a number of existing structures. Broadly, the aim has been better to represent data arising from all assembly workflows. Until our redevelopment work, we had focused our data structures upon traditional clone-based assembly workflows. Given the variety of approaches now in use for assembly, this traditional view introduces increasing limitations in supporting new assembly data and places constraints and complexity upon submitters and consumers of all but traditional assemblies. Our new data structures also support more fluid global exchange of assembly data with our INSDC partners (http://www.ebi.ac.uk/ena/about/standards-genome-assembly-submission).

### Assembly submissions

One of the major challenges in supporting genome assembly submissions is that for each assembly a number of distinct data types (such as reads, contigs, chromosomes and annotation) are provided in different combinations. On top of this, varying requirements for programmatic versus interactive submission tools across submitters and between data types within a submission require a system in which many permutations and combinations of options must be offered. Over the year, we have undertaken a major reworking of our services in this area and have rolled out improvements and new services. This process is expected to reach completion at around the beginning of 2014, from which time major changes are not expected.

Starting our development programme in late 2012, we launched a programme of frequent incremental improvements of ENA assembly submission services to rise to the challenges stated above. The work has also involved the introduction of support for submissions of new types of assembly, most notably genomes consisting of a mixture of contigs and scaffolds built from short reads. The contigs and scaffolds in these genome submissions typically arrive mixed in the same FASTA files without a formal separation of contigs and scaffolds, whereas scaffolds in the more traditional clone-based assemblies are submitted as AGP files containing contig placement and gap information. The new submission system gives submitters the opportunity to choose the appropriate route for a given dataset and at the same time prepares the submitted data for back-end processing pipelines. For interactive submissions, we have made increasing use of the Webin submission web application to guide our submitters through the submission process. Part of the work here has been to merge distinct codebases that support the Webin application and to provide a single application with uniform interface for all components of an assembly submission. Concurrently, we have developed a new and fully automated high-throughput genome assembly processing pipeline, which receives data through Webin and launches internal data processing.

An important part of our development process has been to improve our documentation and to commit to keeping it up-to-date as we roll out changes. The documentation is available at http://www.ebi.ac.uk/ena/about/genome_assembly_submissions. In addition, we have documented our requirements for assembly data at http://www.ebi.ac.uk/ena/about/standards-genome-assembly-submission.

### Introduction of assembly_gap feature

Genome assemblies comprise a number of possible layers of information including reads, contigs, scaffolds and chromosomes. The traditional ENA data model, reflecting the tiered clone-based assembly strategy, has traditionally required submitters to submit data for all layers of assembly. However, it has become increasingly apparent that most assembly tools in use no longer yield assembly intermediates. More typical are data which have been assembled directly into the highest level of assembly, with no intermediate steps, requiring significantly more work to split the assembled scaffold with gaps into component contigs and then build the scaffolds based on the contig records. Consequently, preparing the multi-layer data and processing and loading these multi-level assemblies has required significant additional work for both submitters and ENA staff over single-layer assemblies.

To overcome this problem and reduce the complexity of multi-layer genomes, INSDC has introduced the assembly_gap feature in 2013. This has given submitters the opportunity to report their gap information directly on all sequence records (as opposed to those in the CONstructed data class alone). Further information, including the definition and usage of the assembly_gap feature is available in the INSDC Feature Table Definitions (http://www.insdc.org/documents/feature-table). In our new genome assembly submission system, we have extended this flexibility to allow users to select a minimum gap length for the automatic creation of assembly_gap features. Our system builds an assembly_gap feature for all gaps at, or greater than, the given length with no indicated gap information. For example, if the submitter indicates that the minimum gap length for an assembly is 20, we create assembly_gap feature for all the stretches of ‘N’ bases with the annotated length set to ‘equal or more than 20’.

### Discovery and retrieval of assembly information

**Figure 2.**
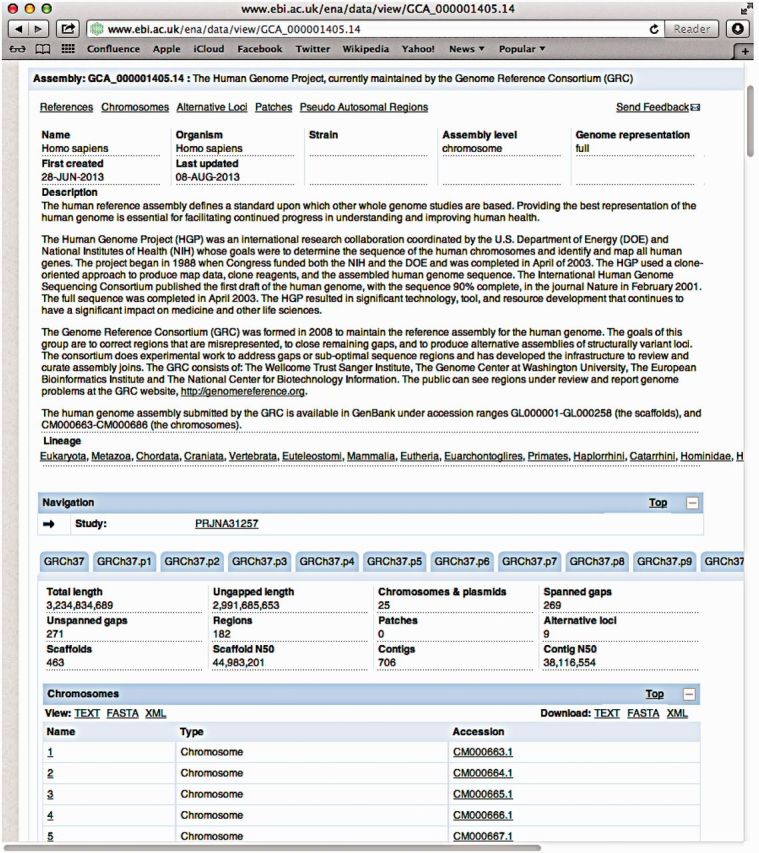
The figure provides a screen shot of an assembly record made visible following a typical search in which the user clicked the ‘Advanced Search’ link from the homepage, checked the ‘assembly’ domain radio button, searched for human genome using organism name ‘human’ and selected GCA_000001405.14 from the listed results. This page provides an assembly description, assembly statistics for all the assembly versions, links to all chromosomes, alternative loci, patches and pseudoautosomal regions in the assembly and references.

There are several ways in which users can retrieve assembly data from ENA. If the accession is known, an assembly record can be viewed directly using the URL http://www.ebi.ac.uk/ena/data/view/<ACCESSION>, e.g. http://www.ebi.ac.uk/ena/data/view/GCA_000001405.14 (see Figure 2). The assembly domain within the ENA Advanced Search service also allows users to find assembly data based on selected fields, such as the organism name and strain designation, the submitters’ given title, name and description for the assembly and by study and entry record accessions. Genome assembly pages link to project records, WGS sets (where available) and any assembled chromosome and organelle sequence records. We are currently working to improve the flexibility of assembly data retrieval and expect these improvements to be available within the first half of 2014.

## FURTHER MAJOR DEVELOPMENTS

### Compression

During the year, we have advanced our technology and services for efficient compression of sequence read data. Specifically, the CRAM software package has progressed to version 2.0 in June 2013 (published openly from http://www.ebi.ac.uk/ena/about/cram_toolkit). CRAM provides format and API support for both lossless and data-reduced quality scale compression. The format can be used as a replacement for BAM in many cases. Indeed a number of our users’ pipelines are undergoing this transition and decisions about the extent of data reduction and choice of reduction model, community decisions for which the discussion continues, can be made separately from the engineering programme of work (10). We have also launched the CRAM Reference Registry, which provides support for CRAM where references do not (yet) exist in ENA (see http://www.ebi.ac.uk/ena/about/cram_reference_registry). Finally, we accept CRAM-format data submissions into ENA and are working towards providing CRAM format data as an output format across ENA read data.

### Submissions

ENA accepts sequence data submission through the Webin service including raw reads, assembled sequences, genome assemblies, functional annotation and associated sample and study information. Both interactive and programmatic submission routes are supported. New submitters are advised to contact datasubs@ebi.ac.uk for advice. Submission instructions are presented at http://www.ebi.ac.uk/ena/about/submit_and_update.

During 2013, in addition to the improvements relating to assembly data submissions described above, we have advanced other areas of the Webin submission application. Improvements include new sample checklist additions, more consistent support for annotated sample records and general usability improvements and modifications to recognize pre-existing sample data from the EBI central BioSamples Database (11). ENA has also created new and updated online tutorials and conducted extensive submissions training within and outside the EBI.

### Search services

Data in ENA can be searched using annotation and sequence similarity search services, available from the ENA home page (http://www.ebi.ac.uk/ena/). Both free and structured text searches are supported across ENA data classes, covering the most important fields associated with studies, samples, reads, sequences, assemblies, taxa, markers and other objects of interest. The ENA sequence similarity search service supports rapid in-memory discovery of assembled sequences.

**Figure 3.**
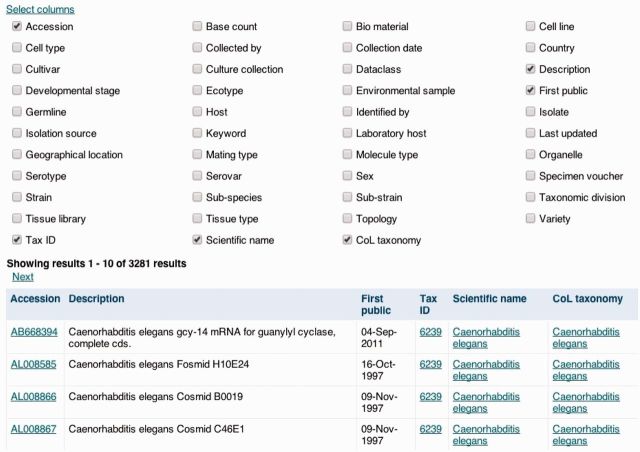
A result table containing selected fields for *Caenorhabditis elegans* results from the sequence domain.

In 2013, ENA made significant improvements to the Advanced Search service (http://www.ebi.ac.uk/ena/data/warehouse/search) that include support for report tables, in which user-selected fields are reported for a given query result set, providing customizable slices across ENA content (see Figure 3). Further major changes to advanced search include improvements to indexing where a systematic review of field name usage has been carried out, followed by a dictionary-based consolidation of these into common searchable fields. This includes consolidation of sample-related fields such as ‘strain’, ‘strain_name’, strain_ID’, etc. (http://www.ebi.ac.uk/ena/data/warehouse/search?portal=sample) and of functional annotation relating to marker loci such as ‘COI’, ‘CoxI’, ‘CO1’, etc. (http://www.ebi.ac.uk/ena/data/warehouse/search?portal=marker).

## FUNDING

ENA is developed and maintained with support from the European Molecular Biology Laboratory, the Seventh Framework Programme of the European Union under i4Life [261555], Micro-B3 [287589], ESGI [262055] and BASIS [242006] and the UK Biotechnology and Biological Sciences Research Council under Metagenomics Portal [BB/I02612X/1] and RNA Central [BB/J019321/1]. Funding for open access charge: European Molecular Biology Laboratory.

*Conflict of interest statement*. None declared.
